# Comparison of 3D and 4D Monte Carlo optimization in robotic tracking stereotactic body radiotherapy of lung cancer

**DOI:** 10.1007/s00066-014-0747-5

**Published:** 2014-09-20

**Authors:** Mark K.H. Chan, Rene Werner, Miriam Ayadi, Oliver Blanck

**Affiliations:** 1 Department of Clinical Oncology, Tuen Mun Hospital, Hong Kong (S.A.R), China; 2 Department of Computational Neuroscience, The University Medical Center Hamburg–Eppendorf, Hamburg, Germany; 3 Department of Radiation Oncology, Léon Bérard Cancer Center, Lyon, France; 4 Department of Radiation Oncology, University Clinic of Schleswig–Holstein, Lübeck, Germany; 5 CyberKnife Center Northern Germany, Güstrow, Germany

**Keywords:** Optimization, Organs at risk, Dose, CyberKnife, Deformable image registration, Optimierung, Risikoorgane, Dosis, CyberKnife, Deformierbare Bildregistrierung

## Abstract

**Purpose:**

To investigate the adequacy of three-dimensional (3D) Monte Carlo (MC) optimization (3DMCO) and the potential of four-dimensional (4D) dose renormalization (4DMC_renorm_) and optimization (4DMCO) for CyberKnife (Accuray Inc., Sunnyvale, CA) radiotherapy planning in lung cancer.

**Materials and methods:**

For 20 lung tumors, 3DMCO and 4DMCO plans were generated with planning target volume (PTV_5 mm_) = gross tumor volume (GTV) plus 5 mm, assuming 3 mm for tracking errors (PTV_3 mm_) and 2 mm for residual organ deformations. Three fractions of 60 Gy were prescribed to ≥ 95 % of the PTV_5 mm_. Each 3DMCO plan was recalculated by 4D MC dose calculation (4DMC_recal_) to assess the dosimetric impact of organ deformations. The 4DMC_recal_ plans were renormalized (4DMC_renorm_) to 95 % dose coverage of the PTV_5 mm_ for comparisons with the 4DMCO plans. A 3DMCO plan was considered adequate if the 4DMC_recal_ plan showed ≥ 95 % of the PTV_3 mm_ receiving 60 Gy and doses to other organs at risk (OARs) were below the limits.

**Results:**

In seven lesions, 3DMCO was inadequate, providing < 95 % dose coverage to the PTV_3 mm_. Comparison of 4DMC_recal_ and 3DMCO plans showed that organ deformations resulted in lower OAR doses. Renormalizing the 4DMC_recal_ plans could produce OAR doses higher than the tolerances in some 4DMC_renorm_ plans. Dose conformity of the 4DMC_renorm_ plans was inferior to that of the 3DMCO and 4DMCO plans. The 4DMCO plans did not always achieve OAR dose reductions compared to 3DMCO and 4DMC_renorm_ plans.

**Conclusion:**

This study indicates that 3DMCO with 2 mm margins for organ deformations may be inadequate for Cyberknife-based lung stereotactic body radiotherapy (SBRT). Renormalizing the 4DMC_recal_ plans could produce degraded dose conformity and increased OAR doses; 4DMCO can resolve this problem.

**Electronic supplementary material:**

The online version of this article (doi: 10.1007/s00066-014-0747-5) contains supplementary material, which is available to authorized users.

## Abstract (de)

**Hintergrund:**

Untersucht wurde die Angemessenheit einer dreidimensionalen (3-D) Monte-Carlo(MC)-Optimierung (3DMCO) und das Potenzial von vierdimensionaler (4-D) Dosisrenormierung (4DMC_renorm_) und -optimierung (4DMCO) für die CyberKnife-Bestrahlungsplanung von Lungentumoren.

**Methoden und Materialien:**

3DMCO- und 4DMCO-Pläne wurden für 20 Lungentumoren erstellt, wobei das Planungszielvolumen (PTV_5 mm_) als makroskopisches Tumorvolumen (GTV) plus 5 mm unter der Annahme definiert wurde, dass 3 mm zur Berücksichtigung des Trackingfehlers (PTV_3 mm_) und 2 mm für residuale Organdeformationen (PTV_2 mm_) ausreichen. Verschrieben wurden 60 Gy/3 Fraktionen auf mindestens 95 % des PTV_5 mm_. Für jeden 3DMCO-Plan wurde eine 4-D-MC-Dosisberechnung (4DMC_recal_) durchgeführt, um die dosimetrischen Auswirkungen von Organdeformationen zu beurteilen. Die 4DMC_recal_-Pläne wurden zum Vergleich mit den 4DMCO-Plänen auf 95 %ige Abdeckung des PTV_5mm_mit der verschriebenen Isodosis renormiert (4DMC_renorm_). 3DMCO wurde als angemessenen angesehen, wenn für den 4DMC_recal_-Plan ≥ 95 % des PTV_3mm_60 Gy erhielten und für die Risikoorgane (OAR) gegebene Dosisgrenzwerte eingehalten wurden.

**Ergebnisse:**

3DMCO war für 7 Läsionen mit < 95 % PTV_3 mm_-Dosisabdeckung unzureichend. Organdeformationen führten im Vergleich von 4DMC_recal_ mit 3DMCO zudem zu niedrigeren OAR-Dosiswerten. Eine Renormierung der 4DMC_recal_-Pläne könnte dann zu Überschreitungen der OAR-Toleranzen für zumindest einige 4DMC_renorm_-Pläne führen. Die Dosiskonformität der 4DMC_renorm_-Pläne war geringer als die der 3DMCO- und 4DMCO-Pläne. Im Vergleich zu 3DMCO und 4DMC_renorm_ führte 4DMCO nicht immer zu einer Dosisreduktion in den OAR.

**Schlussfolgerungen:**

3DMCO mit 2 mm Sicherheitssaum für Organdeformationen kann unangemessen für die CyberKnife-basierte Lungen-SBRT sein. Die Renormierung der 4DMC_recal_-Pläne kann zu reduzierter Dosiskonformität und erhöhten Risikoorgandosen führen. 4DMCO kann dieses Problem lösen.

Real-time tumor tracking can effectively mitigate respiration-induced organ motion effects during stereotactic body radiotherapy (SBRT) of lung cancer [1, 2]. While the treatment accuracy of dedicated SBRT systems such as CyberKnife (Accuray Inc., Sunnyvale, CA) appears to be very high ( < 3 mm) [3], residual organ deformations are rarely considered specifically and commonly taken into account by the margin concept, i.e. by increasing the planning target volume (PTV). To estimate and account for organ deformation, dedicated optimization frameworks on quasi-deforming patient geometry derived from four-dimensional computed tomography (4DCT), commonly known as 4D optimization (4DO), have been developed on various platforms [4–6]. Theoretically, 4DO can reduce the PTV margin, or for non-real time tracked SBRT, the internal target volume (ITV) margin. This is accomplished by taking advantage of the differential motions to optimize dose distribution explicitly, instead of using worst-case margins that potentially limit dose escalation in conventional three-dimensional (3D) optimization.

For lung SBRT, 4DO requires heterogeneity corrections which can be augmented by Monte Carlo (MC) simulations of particle interactions, forming a 4DMC optimization (4DMCO) framework that should result in accurate estimations of dose depositions in an environment of varying tissues densities. Long computing times and a lack of clinical validation remain the primary current impediment to shifting clinical practice from 3DMC optimization (3DMCO) to 4DMCO.

In our previous study of robotic-based lung SBRT using deformable image registration (DIR) for 4D dose accumulation, we showed that organ deformation and the delivery mechanics caused relatively small but noticeable variations in doses to the gross tumor volume (GTV) and normal tissues. Tissue heterogeneities represented the major contribution to the dosimetric errors in direct 4DO [7, 8]. The question now remains whether the organ deformations and the treatment delivery mechanics could be ignored altogether. We hypothesize that MC optimization on static planning CTs (3DMCO) with an adequate safety margin around the GTV to account for residual organ deformations appears to be a reasonable compromise between treatment planning accuracy and efficiency. While 3DMCO can save substantial planning time compared to calculating and deforming the dose distributions in different breathing geometries, the treatment planning accuracy has yet to be proven.

For CyberKnife 3DO, the commonly used PTV margins are 5 mm. Although some studies using 2–3 mm PTV margins also resulted in excellent local tumor control rates, these had very high biological effective doses (BEDs) due to lack of realistic MC dose calculation [9–12]. The general tracking error for CyberKnife has been estimated from treatment logs, resulting in a considerable disparity between clinical treatment accuracies of 1.5–6.9 mm [9, 13, 14]. At our institute, a total PTV margin of 5 mm (PTV_5 mm_) is used in all SBRT plans, regardless of tumor size and location. This 5 mm is made up of a 3 mm margin (PTV_3 mm_) for the tracking error [9, 13, 14] and a 2 mm margin (PTV_2 mm_) accounting for organ deformations [15]. With a 4DMC patient simulation we are now seeking evidence that a 2 mm margin for organ deformation is adequate to ensure sufficient dose coverage (e.g. 95 %) of the PTV_3 mm_ in our 3DMCO strategy. We also investigate the adequacy of renormalizing the prescribed isodose level after 4DMC dose calculation to achieve the desired target dose coverage. Such a 4DMC dose renormalization strategy has the advantage that the 4DMC dose calculation and accumulation only have to be done once, instead of entering into an optimization loop. Whether such a 4DMC dose renormalization strategy will yield a dose distribution comparable to 4DMCO is of interest, as it would directly influence the planning practice. Furthermore, we aim to investigate the potential of 4DMCO for improving overall plan quality.

## Materials and methods

### Patient data and 4DCT simulation

Twenty peripheral lung tumor patients who were deemed surgically inoperable or refused surgery were treated with CyberKnife using the Synchrony Respiratory Tracking System (Accuray) combined with either fiducial tracking (fiducial-based target tracking) or XSight Lung Tracking (fiducial-free target tracking; Accuray). These tumors comprised eight upper-lobe, four middle-lobe and eight lower-lobe peripheral tumors sized between 0.62 and 77.4 cm^3^ (median 11.6 cm^3^). For each patient, 4DCT simulation was performed under free breathing conditions on a GE Light Speed 64-slice CT (GE Healthcare, Little Chalfont, UK). Details of the 4DCT acquisition and reconstruction protocol can be found in our previous publication [7]. Each 4DCT dataset consisted of ten equally time-binned 3DCT images of a single breathing cycle (0–90 %), with 0 and 50 % generally corresponding to the end-inspiration and end-expiration phases, respectively; 30 and 70 % corresponded to the mid-ventilation phase. All 3DCT images were transferred to the CyberKnife treatment planning system MultiPlan (Accuray Inc.) for 3D MC and 4D MC treatment planning.

### Treatment planning, evaluation and 4D dose computation

For all cases, the end-expiration 3DCT images of the 4DCT dataset were used as the primary planning CT and for defining the GTV and other critical structures as per international guidelines [16, 17]. The GTV was expanded isotropically by a 3 mm margin for potential tracking error compensation (PTV_3 mm_) and by further a 2 mm for potential organ deformation compensation (PTV_5 mm_). As in the Radiation Therapeutic Oncology Group (RTOG) 0236 protocol [17], no margin was added for presumed microscopic disease (i.e. the GTV and the clinical target volume, CTV, are identical). The prescribed dose was three fractions of 20 Gy with at least 95 % of the PTV_5 mm_ covered by the prescribed dose. No adjustment of dose prescription according to tumor size was made for changing the dose calculation from the equivalent path length (EPL) correction-based method to the MC method. With this schedule, the dose to 2 % (D_2 %_) was limited to 18 Gy for the spinal cord, for the trachea and the main bronchus to 30 Gy, for the esophagus to 21 Gy and for the heart to 36 Gy. The bilateral lungs were allowed 20 Gy (converted to 2 Gy fractions by the linear-quadratic, LQ, model with α/β = 3 Gy) to less than 31 % of the total volume (i.e. V_20 Gy3_ ≤ 31 %) [18].

CyberKnife treatment planning as per our in-house protocol was first performed by 3DMCO on the primary CT and then by 4DMCO using the full 4DCT dataset. Dose distributions were calculated by the MC method [19] at 0.5 % relative statistical uncertainty, except during the 4DO, for which a larger statistical uncertainty level of 4 % was set to ease the computing demand and reduce the optimization time. The 4DO was performed with the 4D planning module of MultiPlan. Technical details of the 4D planning module were described by West et al. [6] and Schlaefer et al. [20], while the specific details of its implementation at our institute can be found in our previous publications [7, 8]. Briefly, the 4D planning involved (1) registration of each phase of the 4DCT to the primary CT through rigid alignment of the tumor (XSight Lung Tracking) or the fiducials’ position (fiducial tracking), representing the motion compensation of the beam geometries during treatment, (2) construction of a B–spline DIR model from each 4DCT phase to the next, representing residual uncompensated organ deformations and (3) the 4D dose calculation on each individual 4DCT phase, including a weighting function for dose summation for each voxel of the primary CT based on the voxel positions in the 4DCT derived from the DIR vector fields. For 4DMCO, steps 1 and 2 were performed once, while the 4D dose calculation in step 3 was repeated multiple times during sequential optimization [21] of the treatment beam set.

For each patient, a 4DMC dose calculation using the same motion compensation and patient deformation models as described above in 4DMCO was also performed to recalculate the 3DMCO plan, resulting in the 4DMC_recal_ plan. The 3DMCO plan was considered adequate if the 4DMC_recal_ plan fulfilled the protocol dose requirements and constraints for the PTV_3 mm_ (i.e. the PTV accounting for tracking error only) and other critical structures. Subsequently, each 4DMC_recal_ plan was renormalized to 60 Gy to at least 95 % of the PTV_5 mm_ to produce the 4DMC_renorm_ plan. We evaluated this 4D dose renormalization strategy as an effective and safe means to account for the effect of organ deformation by comparing the plan quality with the 4DMCO plan and the original 3DMCO plan.

### Treatment plan evaluation

Plan quality was assessed based on various dosimetric and radiobiological indices. Dose indices, including the dose to 98 % (D_98 %_) as the near-minimum dose; the volume of the GTV, PTV_3 mm_ and PTV_5 mm_ receiving 60 Gy (V_60 Gy_); dose to 2 % (D_2 %_) as the near-maximum dose to the cord, heart, esophagus, trachea/main bronchus and the V_20 Gy3_ and mean lung normalized total dose to 2 Gy fractions (MLNTD) of the lung were evaluated. Dose conformity, defined$(TV\times PIV)/TV_{PIV}^{2}$ by the new conformity index (nCI) [22] as, where TV is the target volume (i.e., PTV_5 mm_), PIV is the volume covered by the prescription isodose line and TV^2^_PIV_ is the volume of the PTV_5 mm_ covered by the prescription isodose line, was compared between the 3DMCO, the 4DMC_renorm_ and the 4DMCO plans. The ideal value of nCI is 1 and smaller nCI values indicate better dose conformity. The radiobiological effect of nonuniform irradiation of the PTV_3 mm_ was assessed by the generalized biological uniform dose (gBEUD) and the tumor control probability (TCP). The gBEUD was calculated by

1 $$ gBEUD={{\left( \sum\limits_{i=1}^{N}{{{v}_{i}}{{\left( EQD{{2}_{i}} \right)}^{a}}} \right)}^{{1}/{a}\;}} $$

where EQD2_*i*_ denotes the dose converted to standard 2 Gy equivalent dose fractions in the *i*th dose–volume histogram (DVH) bin of the PTV_3 mm_; *v*_*i*_ is the fraction of the PTV_3 mm_ receiving EQD2_*i*_; *N* is the number of DVH bins and *a* is the endpoint-dependent fitting parameter which is set to -10, a value representative of rapidly dividing tumor cells. In this work, the TCP was calculated by [23]

2 $$ TCP=\prod\limits_{i=1}^{N}{{{e}^{[BED{{10}_{i}}-c*L-TCD50]/k}}\div \left( 1+{{e}^{[BED{{10}_{i}}-c*L-TCD50]/k}} \right)} $$

where *i* indicates the *ith* voxel of the tumor; BED10 is the biological effective dose calculated with *α/ β *= 10 Gy; *c* is a constant and *L* is the tumor diameter for adjusting the effective dose according to the tumor size; TCD50 is the dose required to achieve 50 % local control and *k* is a fitting constant that is equal to 25 divided by the slope of the TCP curve at a dose equal to TCD50. With the model parameters for c = 10 Gy/cm, TCD50 = 0 Gy and L = 31 Gy, optimized to provide a best fit to the outcomes of 504 stage I non-small cell lung cancer treated by SBRT with total doses of 24–64 Gy in 1–15 fractions [23], eq. 2 can be used to predict the probability of 2-year local control rates. In addition, the complication probability of radiation-induced pneumonitis grade 2 and above (RP2 +) was calculated according to Borst et al. [24] for each plan.

### Statistical analysis

Statistical comparisons were based on Mann–Whitney U tests between two different planning methods (e.g. 3DMCO vs. 4DMCO) and Kruskall–Wallis tests of multiple planning methods (e.g. 3DMCO vs. 4DMC_recal_ vs. 4DMC_renorm_ vs. 4DMCO) using the MATLAB Statistical Toolbox (MathWorks Inc., Natick, MA, USA). Differences were considered significant where *p* < 0.05.

## Results

### Tumor site

**Fig. 1:**
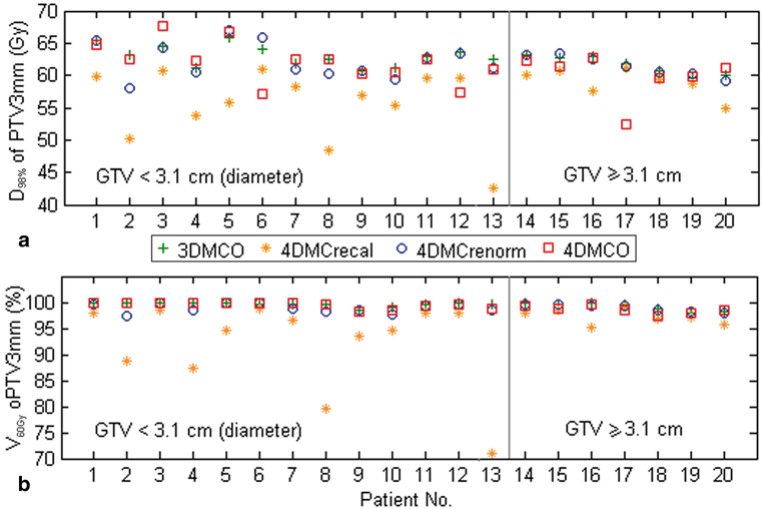
Comparisons of dose to 98 % (D_98 %_) of the planning target volume plus 3 mm (PTV_3 mm_) and the percent volume of PTV_3 mm_ receiving at least prescription dose 60 Gy (V_60 Gy_) obtained by the 3DMCO, 4DMC_recal_, 4DMC_renorm_ and 4DMCO plans in 20 patients, ordered in terms of increasing gross tumor volume (GTV) size. The vertical line shows division of GTV into tumors of diameter < 3.1 cm and ≥ 3.1 cm. See text for descriptions of the different plans

Results of the tumor analysis are summarized in Table 1. Over all patients, the percent volumes of PTV_5 mm_, PTV_3 mm_ and GTV receiving 60 Gy (i.e. V_60 Gy_) decreased by 9.9 ± 8.2 % (one standard deviation, SD; range 1.1–32.0 %), 5.5 ± 7.3 % (0.4–28.6 %) and 0.9 ± 3.1 % (0.0–13.8 %), respectively, from the 3DMCO to the 4DMC_recal_ plans (all *p*-values < 0.05). If a 2 mm margin was assumed for residual organ deformation, 3DMCO failed to maintain 95 % dose coverage of the PTV_3 mm_ in seven plans, with 71.1–94.7 % coverage in the 4DMC_recal_ plans. Furthermore, the impact of organ deformation differed with tumor size, as indicated in Fig. 1. The differences in V_60 Gy_ and D_98 %_ between the 3DMCO and the 4DMC_recal_ plans were less than − 4.6 % (mean = − 1.9 %) and − 5.3 Gy (mean = − 2.7 Gy), respectively, for tumors of diameter ≥ 3.1 cm, but became as much as − 28.6 % (mean = − 7.5 %) and − 20.0 Gy (mean = − 7.5 Gy), respectively, for tumors < 3.1 cm in diameter. The negative signs indicate that V_60 Gy_ and D_98 %_ obtained from 4DMC_recal_ were lower than from 3DMCO (*p*-values < 0.05). Likewise, the 4DMC_recal_ plans showed − 15.7 % and − 7.8 % lower gBEUD of the PTV_3 mm_ for tumors < 3.1 cm and ≥ 3.1 cm in diameter than the original 3DMCO plans, respectively. Nonetheless, the TCP was hardly impacted when the BED10 exceeded 105 Gy_10_ [25]. For example, the TCP decreased by just 1.2 % for a maximum reduction in gBEUD of 85.3 Gy. The mean TCP values were above 99.5 % in both the 3DMCO and the 4DMC_recal_ plans.

**Table 1:** Summary of dosimetric and radiobiological results for 20 patients

	3DMCO	4DMC_recal_	4DMC_renorm_	4DMCO	p-value
*PTV*_*5* *mm*_					
nCI	1.23 ± 0.09		1.42 ± 0.21	1.32 ± 0.21	*p* 0.01^a^ *p* = 0.04^b^ *p* = 0.03^c^
D_98 %_ (Gy)	57.9 ± 1.2	51.9 ± 4.8	57.7 ± 5.7	58.4 ± 5.5	
V_60 Gy_ (%)	95.7 ± 0.5	85.8 ± 8.2	95.3 ± 0.3	95.4 ± 0.5	
*PTV*_*3mm*_					
D_98 %_ (Gy)	62.5 ± 1.7	56.7 ± 4.9	62.0 ± 2.4	61.3 ± 3.3	
V_60 Gy_ (%)	99.4 ± 0.7	93.9 ± 7.2	98.9 ± 0.8	99.2 ± 0.8	
gBEUD (Gy_10_)	194.7 ± 12.2	169.4 ± 27.5	195.6 ± 22.1	196.4 ± 16.2	
TCP (%)	99.9 ± 0.1	99.7 ± 0.3	99.9 ± 0.1	99.9 ± 0.1	
*Spinal cord*					
D_2 %_ (Gy)	9.9 ± 5.1	9.6 ± 4.9	10.6 ± 5.4	10.0 ± 6.0	*p* = 0.60^d^
*Esophagus*					
D_2 %_ (Gy)	8.1 ± 5.6	7.9 ± 5.7	8.6 ± 6.1	8.3 ± 5.5	*p* = 0.97^d^
*Heart*					
D_2 %_ (Gy)	9.0 ± 5.5	8.5 ± 5.1	9.3 ± 6.0	9.9 ± 6.5	*p* = 0.95^d^
*Trachea*					
D_2 %_ (Gy)	7.2 ± 5.9	6.9 ± 5.7	7.5 ± 6.1	7.9 ± 7.1	*p* = 0.97^d^
*Lung*					
V_20 Gy3_ (%);	10.3 ± 7.7	9.8 ± 7.8	10.8 ± 8.2	10.3 ± 7.5	*p* = 0.96^d^
MLNTD (Gy_3_)	10.1 ± 8.0	9.8 ± 8.7	11.4 ± 9.4	10.5 ± 8.0	*p* = 0.89^d^
NTCP (%)	17.7 ± 24.0	17.2 ± 23.7	21.7 ± 28.7	18.8 ± 24.0	*p* = 0.74^d^

Values = mean ± 1 standard deviation (SD). 3DMCO and 4DMCO represent doses optimized on 3D and 4DCT images, respectively. 4DMC_recal_ represents doses obtained from recalculating the 3DMCO plan on the 4DCT images and 4DMC_renorm_represents 4DMC_recal_ subsequently renormalized to achieve 95 % prescription dose coverage of the planning target volume (PTV) plus 5 mm

^a^3DMCO vs. 4DMC_renorm_

^b^3DMCO vs. 4DMCO

^c^4DMCO vs. 4DMC_renorm_

^d^3DMCO vs. 4DMC_recal_ vs. 4DMC_renorm_ vs. 4DMCO

*PTV*_*3* *mm*_ *and PTV*_*5* *mm*_ PTV with 3 mm and 5 mm margin, respectively; *nCI* new conformity index; *D*_*x*__*%*_ dose to *x* % volume of organs; *V*_*xGy*_ percent volume of organ receiving *x* Gy; *MLNTD* mean lung normalized total dose; *TCP* tumor control probability; *NTCP* normal tissue complication probability; *gBEUD* generalized biological equivalent uniform dose. (Note: *Gy*_*x*_ indicates the total dose normalized to 2 Gy using *α/β* of *x* Gy)

For the 4DMC_renorm_ and 4DMCO plans, the V_60 Gy_ and D_98 %_ of PTV_3 mm_ were found to be 98.9 and 99.2 %, and 62.0 Gy and 61.3 Gy, respectively. The gBEUD was not statistically different between the 3DMCO, the 4DMC_renorm_, and the 4DMCO plans (*p* = 0.181). The estimated TCP values were above 99.0 % in all 4DMC_renorm_ and 4DMCO plans because of the significantly high gBEUD in the 3DMCO plans already.

**Fig. 2:**
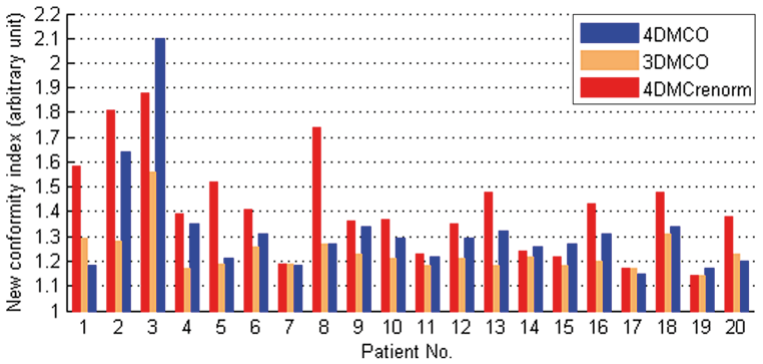
Dose conformity in the planning target volume plus 5 mm (PTV_5 mm_) in terms of the new conformity index (nCI) obtained in the 3DMCO, 4DMC_renorm_ and 4DMCO plans for 20 patients ordered in terms of increasing gross tumor volume (GTV) size. See the main text for descriptions of different the plans

**Fig. 3:**
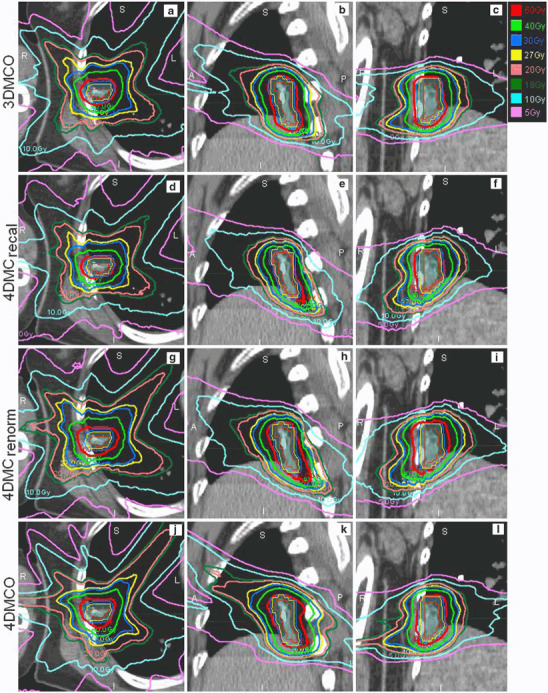
Axial, sagittal and coronal views (from left to right) of dose distributions obtained in the 3DMCO (**a―c**), 4DMC_recal_ (**d―f**), 4DMC_renorm_ (**g―i**) and 4DMCO (**j―l**) plans for one patient with a small gross tumor volume (GTV 4.2 cm^3^). The thin red, amber and blue lines represent GTV, planning target volume plus 3 mm (PTV_3 mm_) and 5 mm (PTV_5 mm_), respectively. See main text for descriptions of the different plans

**Fig. 4:**
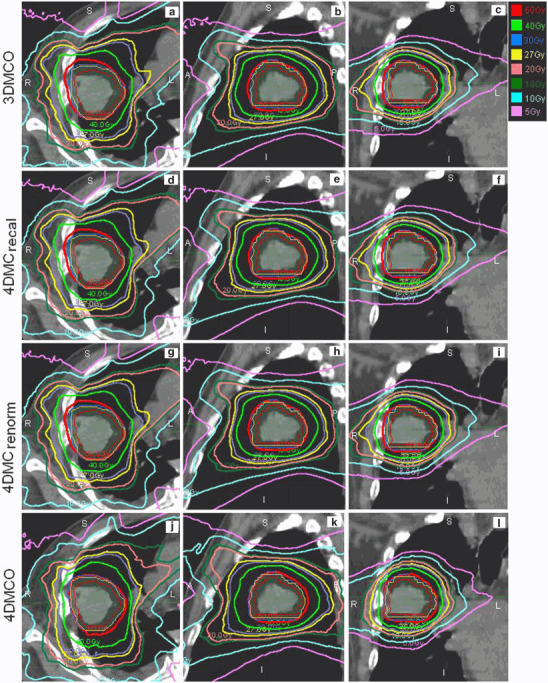
Axial, sagittal and coronal views (from left to right) of the dose distributions obtained in the 3DMCO (**a―c**), 4DMC_recal_ (**d―f**), 4DMC_renorm_ (**g―i**) and 4DMCO (**j―l**) plans for one patient with a large gross tumor volume (GTV 31.7 cm^3^). The thin red, amber and blue lines represent GTV, planning target volume plus 3 mm (PTV_3 mm_) and 5 mm (PTV_5 mm_), respectively. See main text for descriptions of the different plans

Although renormalization represents an efficient means to deal with the effect of organ deformation, it does not necessarily reproduce the conformal dose distribution that would have been expected from the original 3DMCO plan. The nCIs of most 4DMC_renorm_ plans were worse than those of the 3DMCO and the 4DMCO plans (Fig. 2). Statistical results of the multiple pair-wise comparisons of nCIs are given in Table 1. Typical dose distributions obtained in the 3DMCO, 4DMC_recal_, 4DMC_renorm_ and 4DMCO plans are shown for a patient with GTV size of 4.2 cm^3^ in Fig. 3. Note the significant dose reduction of PTV3 _mm_ from the 3DMCO to 4DMC_recal_ plans due to the organ motion and deformations, and the degraded dose conformity from the 3DMCO to 4DMC_renorm_ plans (nCI 1.23 vs. 1.74). The dose conformity of the 4DMCO plan (nCI = 1.27) exhibited inferior conformity posterior to the PTV_5 mm_ compared to the 3DMCO plan. For large GTVs (31.7 cc), the variations in dose conformity were less obvious between plans (Fig. 4). Note the similar dose distributions between plans in the high-dose region and superior dose distribution of the 4DMCO plan in the medium- to low-dose region.

### Critical structures

Table 1 also includes the dosimetric and radiobiological statistics of the critical structures. Due to the close proximity of the tumors to critical organs, two 3DMCO plans exceeded the D_2 %_ constraints for spinal cord and esophagus. For one tumor attached to the spinal column, the cord D_2 %_ was found to decrease to below the dose constraint from 18.6 Gy in the 3DMCO plan to 17.1 and 17.8 Gy in the 4DMC_recal_ and 4DMC_renorm_ plans, respectively. For the other left apex tumor in close proximity to the esophagus, the D_2 %_ esophagus constraint was exceeded by 1.3 Gy in the 3DMCO plan, by 1.8 Gy in the 4DMC_recal_ and 3.2 Gy in the 4DMC_renorm_ plans.

**Fig. 5:**
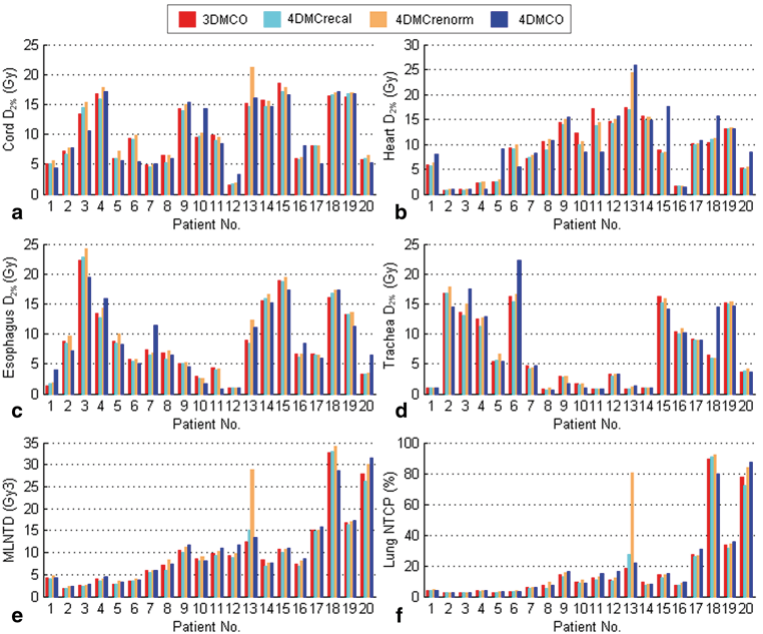
Dosimetric results of critical structures obtained in the 3DMCO, 4DMC_recal_, 4DMC_renorm_ and 4DMCO plans in 20 patients ordered in terms of increasing gross tumor volume (GTV) size. **a―d** Dose to 2 % volumes (D_2 %_) of spinal cord, heart, esophagus, trachea; **e** mean lung normalized total dose (MLNTD); **f** normal tissue complication probability (NTCP) of radiation-induced pneumonitis of grade 2 and above. See the main text for descriptions of the different plans

In comparisons with the 3DMCO plans, the 4MC_recal_ plans resulted, on average, in slightly lower doses to the lung and other critical structures, while the renormalized 4MC_renorm_ plans produced nonsignificantly higher doses to all critical structures (Table 1 and Fig. 5). It is important to note that the 4D renormalization strategy resulted in 5 % of the plans (1 out of 20) exceeding at least one dose constraint that had originally been achieved in the 3DMCO plans (i.e. spinal cord D_2 %_ of patient no. 13, see Fig. 5).

As shown in Fig. 5, the 4DMCO plans did not result in an obvious reduction in the lung dose and showed slightly higher (but nonsignificant) doses to some critical structures (e.g. heart and trachea) compared to the 4DMC_renorm_ plans. Dosimetric comparisons of the lung volume receiving high to low doses (e.g. V_50 Gy3_, V_30 Gy3_, V_20 Gy3_, V_10 Gy3_ and V_5 Gy3_) between the 4DMCO and the 4DMC_renorm_ plans are given in supplementary Fig. 1. In our cohort of 20 patients, the NTCP of normal lung was above 5 % in 14 patients. A clear cutoff of lung NTCP above 5 % was observed for a GTV of size larger than 3.5 cm^3^ Statistical comparisons indicated nonsignificant differences in all evaluated dosimetric and radiobiological parameters between the 3DMCO, 4DMC_renorm_ and 4DMCO plans (all *p*-values > 0.05). Nonetheless, 4DMCO ensures doses to all critical structures within the defined limits that could be exceeded in some 3DMCO and 4DMC_renorm_ plans (Fig. 5).

## Discussion

In real-time motion compensated robotic SBRT of lung cancer, the use of adequate safety margins to compensate for residual tracking errors (e.g. due to irregular organ motion, inaccuracies in correlation and prediction modeling, system latencies or nonrigid organ deformations) appears to be a reasonable compromise between treatment accuracy and treatment planning time. However, implicit accounting for treatment delivery uncertainties in such a complex mode of 4D real-time tracking radiotherapy may put a proportion of patients at risk of receiving a deficient tumor dose, which may negatively impact tumor control and/or normal tissue doses, which might lead to unacceptable toxicities or even treatment-related morbidity. With the commonly used 5 mm PTV margin for robotic SBRT assuming a 3 mm margin to account for tracking imperfection (i.e., PTV_3 mm_), this study found that using an additional 2 mm margin to compensate for the residual organ deformations could cause the prescription dose to encompass less than the desired 95 % of the PTV_3 mm_ in 35 % of patients. Whether this deficient tumor dose coverage was compensated for by smaller tracking errors and hence by the original 3 mm tracking error margin in these patients is unknown and may require further investigation; however dosimetric influences from organ deformations would have simply gone unnoticed from the 3DMCO plans alone without performing a 4D MC dose recalculation (i.e. the 4DMC_recal_ plan). In addition, it was demonstrated that by renormalizing the 4DMC_recal_ plan, one can efficiently retrieve the target dose coverage; however, on the other hand, this could cause the dose constraints of the critical structure to be exceeded. This was indeed observed in one case, where the patient’s spinal cord D_2 %_ was found to be 21.1 Gy and hence beyond our clinical limit of 18 Gy. Furthermore, this approach generally degrades the target dose conformity with respect to 3DMCO plans, which goes against the principles of high-dose robotic SBRT.

Our results match previous estimations of the 3 mm margin for organ deformations [15]. However, the latter study used only a simple approach based on two instances of respiratory geometries at end-inspiration and end-expiration, without accurate MC dose calculation. In contrast to 4D planning studies without motion compensation [25, 26] and previous studies [7, 20], we now present a realistic estimation of dosimetric influences of uncompensated residual organ deformation (e.g. target coverage drop, critical structure dose increase, changes in dose conformity etc.) from a larger patient cohort under real-time robotic tracking. Whenever there is any major violation of the dose constraints of the critical structures in the renormalized (4DMC_renorm_) plan that concerns the treating physicians, the treatment planner is generally made to start over, by using 3DMCO and setting a lower dose limit to this structure as the planning objective or by simply lowering the prescription dose to adapt the NTCP, or, as we presented, by using 4DMCO. The 3D reoptimization approach is nonintuitive and based on the presumption that the resulting reoptimized 3DMCO dose will be lower than the tolerance limit. Whether it is really below the tolerance dose remains unknown unless another 4DMC_recal_ plan is obtained. This may lead to a timely expensive trial and error process, including multiple 4DMC dose calculations, particularly in the case of close proximity to critical structures. The approach to lower the prescription dose to ensure critical structure dose limits may, on the other hand, seriously compromises TCP, which may be significant if the overall 5 mm safety margin does not compensate for all treatment errors and a lower BED10 (e.g. lower or close to 105 Gy_10_) is used [25]. Furthermore, many local recurrences may be explained when calculating the 4D MC dose and may be avoided in the future with the methods presented here. The last option of rerunning the optimization by 4DMCO represents the most accurate treatment planning approach to gauge the physical feasibility of the specified dose objective under a more realistic environment of dynamic patient geometry; however 4DMCO does not necessarily guarantee that the dose constraints will become achievable in cases where they were not achievable with 3DMCO with static margins.

Our results demonstrate that 4DMCO can effectively achieve target dose conformity and assure normal tissue doses at least as well as 3DMCO and subsequent renormalization after 4DMC dose calculation, yet with the additional advantage of guaranteed target dose coverage. The advantage of 4DMCO over 3DMCO is expected to increase with the margin size required to compensate for organ deformations. While 4DMCO is computationally more expensive than 3DMCO, it may be faster than iterative 3DMCO (i.e. if the worst tracking inaccuracy is always assumed) with additional calculations of 4D dose distribution for verification.

One may argue that 4D dose renormalization is not necessary if the PTV_3 mm_ receives over 95 % coverage as a 2 mm margin compensates for the effects of organ deformations. A similar question is whether the prescription dose should be renormalized at all to ensure adequate dose coverage of the PTV_3 mm_ rather than using the PTV_5 mm_ in cases where the PTV_3 mm_ shows V_60 Gy_ < 95 %, because organ deformations are explicitly compensated by the 4D MC dose calculation. The high complication rate of radiation-induced pneumonitis grade 2 and above (RP2 +) estimated by the model of Borst et al. [24] represents a major concern and may justify the option of renormalizing a (lower) prescription dose to the PTV_3 mm_, as long as the BED is maintained at 105 Gy_10_ and above. This may be particularly important for large tumors, as demonstrated in Fig. 5 and supplementary figure 1.

One limitation to our study is that we could not investigate whether the pattern of breathing motion and its influence on the organ deformation as experienced in the 4DCT will maintain throughout the treatment. In other words, until real-time volumetric imaging is acquired during treatment, it remains questionable whether the margin that is found sufficient to compensate for organ deformations during the 4DCT imaging will still be sufficient in the presence of motion variability, i.e. whether 4DMCO will correctly compensate for organ deformations in all patients in all situations. There are scarce data in the literature reporting changes in lung tumor volume during SBRT. In one study of lung SBRT, Matsugi et al. [27] observed nonsignificant variations of tumor volume during the SBRT sessions. Unfortunately, this study did not report the dosimetric margin needed to account for the random and systematic volume changes. In contrast, significant intrafractional variability of motion patterns has been previously reported in some patients in lung SBRT [28]. Further investigation on the variability of motion amplitudes and the influence on the extent of organ deformation and quality assurance, verification of deformation modeling and 4D dose calculation are warranted.

## Conclusion

In real-time tracking robotic SBRT of lung cancer, 3DMCO treatment planning with the commonly used 5 mm PTV margin may provide inadequate tumor dose coverage if a 2 mm margin is used to compensate for organ deformation. Renormalizing the plans after 4D dose calculation to achieve the desired target coverage could result in degraded target dose conformity and increased normal tissue doses; 4DMCO can resolve this problem.

## Electronic supplementary material

(PDF 17 kb)
