# Correction to: Comparison of 3D and 4D Monte Carlo optimization in robotic tracking stereotactic body radiotherapy of lung cancer

**DOI:** 10.1007/s00066-022-01967-6

**Published:** 2022-06-27

**Authors:** Mark K. H. Chan, Rene Werner, Miriam Ayadi, Oliver Blanck

**Affiliations:** 1 Department of Clinical Oncology, Tuen Mun Hospital, Hong Kong (S.A.R), China; 2 Department of Computational Neuroscience, The University Medical Center Hamburg–Eppendorf, Hamburg, Germany; 3 Department of Radiation Oncology, Léon Bérard Cancer Center, Lyon, France; 4 Department of Radiation Oncology, University Clinic of Schleswig–Holstein, Lübeck, Germany; 5 CyberKnife Center Northern Germany, Güstrow, Germany

**Correction to:**

**Strahlenther Onkol (2015) 191:161–171**

10.1007/s00066-014-0747-5

The article **Comparison of 3D and 4D Monte Carlo optimization in robotic tracking stereotactic body radiotherapy of lung cancer**, written by **Mark K. H. Chan, Rene Werner, Miriam Ayadi, Oliver Blanck**, was originally published Online First without Open Access. After publication in volume 191, issue 2, page 161–171 the author decided to opt for Open Choice and to make the article an Open Access publication. Therefore, the copyright of the article has been changed to © The Authors 2014 and the article is forthwith distributed under the terms of the Creative Commons Attribution 4.0 International License, which permits use, sharing, adaptation, distribution and reproduction in any medium or format, as long as you give appropriate credit to the original author(s) and the source, provide a link to the Creative Commons licence, and indicate if changes were made. The images or other third party material in this article are included in the article’s Creative Commons licence, unless indicated otherwise in a credit line to the material. If material is not included in the article’s Creative Commons licence and your intended use is not permitted by statutory regulation or exceeds the permitted use, you will need to obtain permission directly from the copyright holder. To view a copy of this licence, visit http://creativecommons.org/licenses/by/4.0/.

