# Supplementary material for: Comparison of 3D and 4D Monte Carlo optimization in robotic tracking stereotactic body radiotherapy of lung cancer
Source: Strahlenther Onkol. 2014 Sep 20;191(2):161–71. doi: 10.1007/s00066-014-0747-5 (PMC9205812; doi:10.1007/s00066-014-0747-5)
Supplement: Supplementary file 1 — (PDF 17 kb) [file 66_2014_747_MOESM1_ESM.pdf]

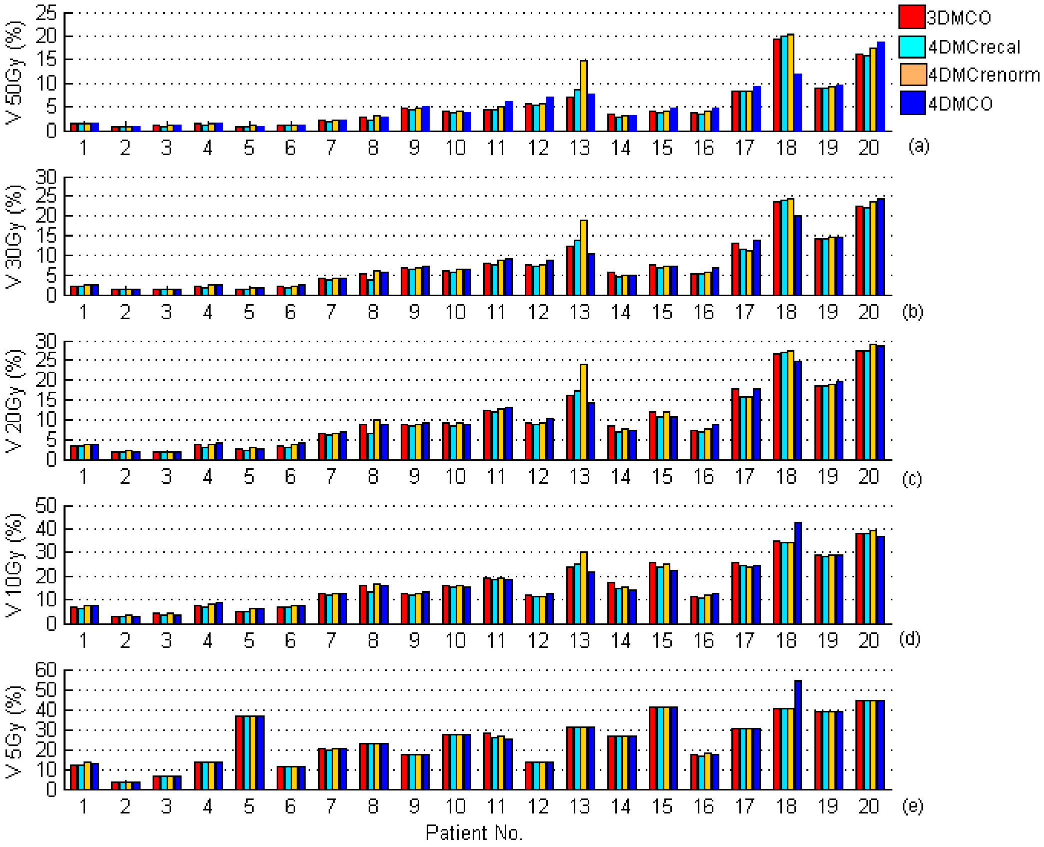

Fig. S1 Bar plot showing the percent normal lung volume receiving (a) 50 Gy, (b) 30 Gy, (c) 20 Gy, (d) 10 Gy, and (e) 5 Gy in the 3DMCO, $4DMC_{recal}$, $4DMC_{renorm}$, and 4DMCO plans in 20 patients ordered in increasing GTV size.
